# How Does Digital Media Search for COVID-19 Influence Vaccine Hesitancy? Exploring the Trade-off between Google Trends, Infodemics, Conspiracy Beliefs and Religious Fatalism

**DOI:** 10.3390/vaccines11010114

**Published:** 2023-01-03

**Authors:** Jiayue Gao, Syed Hassan Raza, Muhammad Yousaf, Amjad Ali Shah, Iltaf Hussain, Aqdas Malik

**Affiliations:** 1 The Bartlett, UCL Faculty of the Built Environment, University College London, London WC1E 6BT, UK; 2 Institute of Media and Communication Studies, Bahauddin Zakariya University, Multan 66000, Pakistan; 3 Centre for Media and Communication Studies, University of Gujrat, Gujrat 50700, Pakistan; 4 Department of Medicine, Health Science Centre, Xi’an Jiaotong University China, Xi’an, 710000, China; 5 Department of Information Systems, Sultan Qaboos University, Muscat 112, Oman

**Keywords:** vaccine hesitancy, COVID-19, digital media, google trends, religious fatalism, infodemics, conspiracy belief, Pakistan

## Abstract

Digital media has remained problematic during COVID-19 because it has been the source of false and unverified facts. This was particularly evident in the widespread misinformation and confusion regarding the COVID-19 vaccine. Past research suggested infodemics, conspiracy beliefs, and religious fatalism as potential threats to public COVID-19 vaccine hesitancy. However, the literature is primarily void of empirical evidence associating demographic attributes with efforts to build vaccine hesitancy. Therefore, this research uses two studies: (Study 1) Google Trends and (Study 2) survey method to provide inclusive empirical insight into public use of digital media during COVID-19 and the detrimental effects of infodemics, conspiracy beliefs, and religious fatalism as they were related to building COVID-19 vaccine hesitancy. Using Google Trends based on popular keywords the public searched over one year, Study 1 explores public digital media use during COVID-19. Drawing on this exploration, Study 2 used a cross-sectional national representative survey of 2120 adult Pakistanis to describe the influence of potential hazards such as infodemics on public vaccine hesitancy. Study 2 revealed that infodemics, conspiracy beliefs, and religious fatalism predict vaccine hesitancy. In addition, gender moderates the relationship between infodemics and conspiracy beliefs and vaccine hesitancy. This implies that there is a dispositional effect of the infodemics and conspiracy beliefs spread digitally. This study’s findings benefit health and other concerned authorities to help them reduce religious fatalism, vaccine hesitancy, and conspiracy theories with targeted communication campaigns on digital media.

## 1. Introduction

The global transmission of the subvariant of Omicron, such as “XBB”, poses a major threat to world health systems. These and similar threats create unprecedented uncertainty and panic [1]. Therefore, to address this question in the current age of digital media, it is significant to explore the influence of infodemics, conspiracy beliefs, and religious fatalism on vaccine hesitancy. WHO defines an infodemic as “an increase in information, including erroneous or misleading information, in digital and physical surroundings during an epidemic of a disease” [2]. The abundance of ‘infodemics’ preceded the rise of COVID-19 infections, and these infodemics became a dominant source of falsehoods that posed a severe threat to public health across the globe [3]. The mixture of accurate and inaccurate information in an infodemic confuses the public about which facts are true and which are false [4,5]. Infodemics are characterized by misinformation, disinformation, and fake news. Misinformation and disinformation are often used interchangeably. However, they have quite different meanings. Misinformation is quite simply false or inaccurate information. The authors do not necessarily have nefarious intentions. Disinformation, on the other hand, is the intentional spread of misinformation with the intention of misleading, deceiving, or confusing [6,7]. There is widespread misinformation and disinformation on social media platforms, and its public availability undermines trust in governmental interventions to counter the pandemic, public health responses, expert guidance, and scientific knowledge necessary to combat COVID-19 [8,9].

In contrast, fake news combines disinformation and misinformation [10]. It presents incorrect and misleading information as news about the origin of the virus and the efficacy of vaccines. Fake news spread by institutions and influencers who are otherwise considered credible and trustworthy plays a substantial role in shaping human behaviors that can lead to poor health outcomes vis-à-vis taking precautionary measures or accepting vaccines [11].

COVID-19 was identified in Wuhan, Hubei Province, China in December 2019 [12,13]. It is caused by the severe acute respiratory syndrome coronavirus 2 (SARS-CoV-2) [14]. Due to the unprecedented and rapid spread of the disease, it was declared the century’s most prevalent public health emergency [14]. Consequently, on 11 March 2020, the World Health Organization (WHO) declared the COVID-19 pandemic. The flood of infodemics amplified this pandemic. Keeping this in mind, WHO Director-General Tedros Adhanom Ghebreyesus, on 15 February 2020, aptly remarked, “We’re not just fighting an epidemic; we’re fighting an infodemic” [15]. As of 11 October 2022, there had been 619,161,228 confirmed cases of COVID-19 worldwide and 6,537,636 global deaths. As of 3 October 2022, 12,723,216,322, vaccine doses had been administered across the globe [16].

The uncertainty created by a pandemic and chronic isolation can lead to panic and psychological distress, such as stress, loneliness, insomnia, depression, and anxiety [17]. Likewise, the misinformation disseminated through social media influences public behavior, creating panic [18]. Information sharing increased exponentially during COVID-19 owing to the pervasive nature of social media in our lives [19]. Moreover, COVID-19 has been referred to as a “digital pandemic” due to the multitude and impact of misinformation circulated on digital platforms. Even before the pandemic, a 2018 study had predicted that the next major breakout would be magnified digitally [20].

It is noted that social media and technological communication advances amplify misinformation’s impact [21]. Research shows that people share falsehoods more than evidence-based information on social media platforms [22,23,24]. In contrast, traditional media, for instance, newspapers, play a positive role in vaccine acceptance. In a recent study, Yousaf et al. [25] found that “the message-consistent effects of media frames manifesting fear (e.g., consequence and uncertainty) and action cues made receivers more supportive of vaccination” (p. 1855). Likewise, it was concluded that fear-oriented messages are the most effective communication strategy in combating vaccine hesitancy [26]. However, there were an estimated 4.2 billion social media users, expected to increase to almost six billion in 2027; therefore, especially during a crisis such as COVID-19, it became the primary source of information sharing, communication, and staying connected regardless of the authenticity of the information shared [27].

A conspiracy theory is generally referred to as “an explanation of historical, ongoing, or future events that cites as a main causal factor a group of powerful persons, the conspirators, acting in secret for their benefit against the common good” (p. 235) [28]. COVID-19 was coupled with a flood of conspiracy theories. These theories hamper the efforts to curb the spread of the virus [26]. A range of conspiracy theories surrounds COVID-19; for instance, the virus was created as a bioweapon [29], it was a source of profits for pharmaceutical companies [30], and a plan to implant microchips for birth control [31]. In the early days of the pandemic, the situation in Pakistan worsened, and the average infection rate rose to 22.6%, indicating exponential growth in the country’s daily infection count from 1000 in May to 6000 in June 2020 [32]. As of 12 October 2022, there had been 1,572,972 confirmed cases of COVID-19, with 30,623 deaths [33]. Understanding people’s belief in conspiracy theories about the coronavirus during the pandemic is significant in many ways. For instance, in countries such as Pakistan, people are highly prone to believing in conspiracy theories concerning health-related issues. For example, Pakistan is one of the two remaining countries worldwide with polio cases (e.g., endemic), largely because of the failure of vaccination campaigns. Many believe the conspiracy theory that the polio vaccine is a ploy designed by the US Central Intelligence Agency to make Muslim men sterile to control the population in Muslim countries [34,35]. A poll in Pakistan revealed that one-third of respondents believes in conspiracy theories related to the COVID-19 pandemic [32].

Despite these intriguing results about misinformation, disinformation, fake news, and conspiracy theories, little research has been conducted on the association between the digital media usage of behavioral patterns, such as vaccine hesitancy. This study fills that gap in the literature by first exploring the public search patterns about the COVID-19 vaccine using Google Trends and the possible adverse outcomes. Secondly, this research examines the major key terms searched during the peak era of COVID-19. We designed a questionnaire to describe the possible link between digital searches for the COVID-19 vaccine and possible detrimental factors such as infodemics. Thirdly, through a nationally representative survey, this research provides evidence to support the claims that search patterns can instill vaccine hesitancy. Lastly, this study examines the role demographics play in assessing how vulnerable certain groups are to the influence of infodemics, conspiracy beliefs, and religious fatalism and whether heightened susceptibility leads to COVID-19 vaccine hesitancy. To this end, this research provides pioneer findings that explore public digital media usage and simultaneously describe public COVID-19 vaccine hesitancy patterns. To do so, this research used two studies: (a) Google Trends and (b) a survey method to answer the rarely studied queries lacking in the literature on how the infodemic of misinformation, disinformation, and fake news found accepting audiences through public digital media usage during the COVID-19 pandemic and led to COVID-19 vaccine hesitancy. The following section sheds greater detail on the implications and exciting findings.

## 2. Literature Review

### 2.1. Background: Digital Media Ecology and Health Behavior

The emergence of the Internet in the last few decades revolutionized communication [36]. The public now has more access to information ranging from the news or events to healthcare decisions [37]. Digital media and the Internet have provided everyone access to diverse sources that were limited to an elite few in the past. Although the growth of resources is a positive aspect of the digitalization of information, it has also significantly increased the chances of spreading misperceptions and negative beliefs [38]. For example, before the Internet, media mainly developed content from official and trusted sources and disseminated reliable information to the public with editorial oversight that ensured that all information was rigorously fact-checked before being released. Many of these media organizations have continued to provide accurate and honest information on the Internet. However, new digital media powered by diverse Internet-based information has changed this information-seeking scenario [39]. For example, everyone can search for particular events on the Internet, but they need help to analyze the quality of information. In the past, information was most often factual and accurate, but the Internet has allowed user-generated content, that mainly contains opinions and analysis rather than facts.

In recent years, particularly during COVID-19, a stream of research has verified that the quality of the information available online has been compromised [40]. In this emerging digital media ecology, people are more vulnerable to misinformation and an overabundance of information that can confuse rather than inform. This is particularly true for healthcare decisions, such as vaccination [40]. Our study reinforces previous research and health authorities that delineated infodemics, conspiracy beliefs, and religious fatalism as critical factors spread through digital media and influencing COVID-19 vaccine hesitancy. People during COVID-19 remained concerned about health hazards and searched for information to help them make informed decisions. Therefore, it is timely to gain insight into how people are using the Internet to search for information on the COVID-19 vaccine.

People from the developing world also need more digital media literacy so they can rely less on unreliable or fake sources when making decisions regarding the COVID-19 vaccine. The following section examines the main literature-oriented factors and presents vaccine hesitancy assumptions.

### 2.2. Hypotheses Development

#### 2.2.1. Impact of Infodemics on Vaccine Hesitancy towards COVID-19

*Washington Post* columnist Rothkopf first used infodemic in 2003 [41]. There are several definitions for it, ranging from “a few facts combined with fear, speculation, and rumor exaggerated and communicated fast globally by new information technology” to “a rapid and far-reaching spread of both factual and erroneous information about something, such as a disease” [42].

It is interesting to note that during the COVID-19 outbreak, the dissemination of false information has considerably increased [43], a few examples being treatments requiring the use of salt water, chlorine, or garlic, dubious reports of lockdowns encouraging panic purchasing, and vaccine-related misinformation generating anxiety among the population, blocking international methods to treat the sickness [44]. Government agencies throughout the globe devised and implemented several initiatives to stop the spread of misinformation, such as advising the public to verify suspicious news via fact-checking websites and highlighting the harmful effects of the behavior through online media [45].

The fake news trend is not new; the word first gained fame during the 2016 US presidential election [46]. p Empirical research has primarily analyzed and investigated the consequences of false news in business, elections, healthcare, and on people’s cognitive abilities [47]. Most of the 57 papers included in a systematic review study (2012–2018) that focused on false health-related news used theoretical frameworks from network science and psychology to analyze immunization for Ebola, and the Zika virus as their primary sources of information [48]. A study of the pre-COVID-19 pandemic literature found that the great majority of relevant research focuses on applying artificial intelligence (AI) methods to detect false news [49].

The current coronavirus (COVID-19) pandemic has brought to light the interconnection of the contemporary globalized globe, where public health concerns may spread far beyond their point of origin owing to the ubiquity of the Internet. Infodemics around the COVID-19 pandemic are harder to control in the current age since social media has established itself as a significant information source. 

In addition to increasing the challenges facing healthcare, social, educational, economic, political, environmental, cultural, and socioeconomic systems around the world, the COVID-19 pandemic outbreak has also given rise to a variety of misinformation epidemics, including rumors, myths, superstitions, conspiracy theories, claims, hoaxes, false misinformation, fake news, mistrust in science during times of crisis, a lack of fact-checking, and misinformation in the form of misleading content [15].

The use of social media as a new platform for disseminating rumors, purposeful misinformation, disinformation, conspiracy theories, and personally motivated narratives to appeal to followers, get attention, and incite fear [50]. Along with the quantity of digital media content, the nature and quality of that exposure are equally important. For example, exposure to violent or graphic imagery, conspiracy theories, or terrible events may cause posttraumatic stress disorder and future apprehension, all of which impair personal functioning. The worldwide public has acquired a preference for news channels due to the mainstream media’s expansion, including electronic, print, and social media. Youth use social media sites such as TikTok, Instagram, YouTube, Twitter, Facebook, and WhatsApp. In the context of the COVID-19 vaccine, much unchecked information has been disseminated. Misleading information about the COVID-19 vaccine threatens vaccine acceptance among the public. For instance, much user-generated content, such as blogs accessible using Google search, provides misleading information about preventing the COVID-19 virus. This misinformation misleads the public by giving some cure-related information, such as gargling with hot water to treat the COVID-19 virus. Moreover, some information argues that the COVID-19 vaccine is unsafe. In this scenario of digitally spread infodemics, we hypothesized that:

**Hypothesis** **1** **(H1)**. *Infodemics positively impact vaccine hesitancy towards COVID-19 (VHC).*

#### 2.2.2. Influence of Conspiracy Beliefs on VHC

Conspiracy theories are associated with fear of vaccines, which corresponds to distrust in science and government around the globe [29,51]. Moreover, conspiracy beliefs are a perilous threat to the credibility of the government, non-government authorities, and international health institutions. The conspiracy beliefs cultivated vis-à-vis conspiracy theories influence the public’s precautionary behavior, such as social distancing or wearing masks to curtail the spread of COVID-19 [52]. During a crisis such as COVID-19, the public’s dependence on media increases many-fold. Nevertheless, social media platforms are susceptible to misinformation, disinformation, fake information, opinionated content without editorial checks, and content that cultivates anti-vaccine attitudes [53,54]. The COVID-19 pandemic has been termed the first social media infodemic (Hao and Basu, 2020). For instance, the widely spread conspiracy theories on social media included COVID-19 vaccination as a tool for birth control, profit for influential pharmaceutical companies, and a bioweapon [55]. The instant literature found a negative correspondence between beliefs and COVID-19-related precautionary measures such as wearing masks and getting vaccinated [56,57].

The previous literature reveals that information overload created due to profuse misinformation corresponds to psychological distress among people [58] and increases vaccine hesitancy towards COVID-19 [59]. As a result, the abundance of the abovementioned content on social media is one of the leading concerns for combatting vaccine hesitancy among segments of the populace. Therefore, in light of a literature review, as mentioned earlier, we hypothesized that conspiracy beliefs positively influence vaccine hesitancy towards COVID-19.

**Hypothesis** **2** **(H2)**. *Conspiracy beliefs positively impact vaccine hesitancy towards COVID-19 (VHC).*

#### 2.2.3. Impact of Religious Fatalism on Vaccine Hesitancy towards COVID-19

Past research identified several psychological and informational factors that can adversely influence health behaviors. However, the literature has also identified religious factors that contribute to determining one’s health behavior. Religious beliefs can determine how individuals anticipate health decisions [60]. The research reported that several religious beliefs can constrain public health responses. These studies noted that religious beliefs such as fatalism can adversely influence health behavior and reduce the public health response. Often, fatalistic views can conflict with the endorsements of health authorities and experts and decrease a person’s positive health response, such as vaccination [61]. Among these religious factors, the literature has identified religious fatalism as the source of developing conflicts and misinterpretations. Religious fatalism is the belief that a person’s health consequence is prearranged or preordained by a greater authority (i.e., divine) and not in the person’s control. Therefore, believers ignore health authorities’ recommendations. Based on this information, we hypothesized that:

**Hypothesis** **3** **(H3)**. *Religious Fatalism positively impacts vaccine hesitancy towards COVID-19 (VHC).*

#### 2.2.4. The Moderating Role of Gender

Demographic variables such as gender, age, and socioeconomic status play a significant role in the intention to take COVID-19-related precautionary measures. For instance, it is assumed that age significantly moderates COVID-19 vaccine hesitancy. Previous studies validate that older adults with low social status are more vulnerable to fake news and conspiracy beliefs [62,63]. Likewise, it is found that females reported more information overload compared to males [64]. Keeping this line of argument in mind, we hypothesized that:

**Hypothesis** **4** **(H4)**. *Gender moderates the relationship between (4a) infodemics and (4b) vaccine hesitancy towards COVID-19.*

## 3. Materials and Methods

### 3.1. Design, Participants, and Procedure

In order to assess the popularity of various COVID-19-related topics for Study 1, we used the Google Trends tool. Google Trends is a web-based service that extracts the popularity of practical search terms in Google search. Through Google Trends, search volume and interest over a period of time across various languages and regions can also be accessed. In recent years, the tool has gained popularity among the research community and has been utilized predominantly to study search patterns, trends, and variations on a given topic. For instance, Nghiem et al. investigated public interest in conservation-related topics over ten years [65]. Many studies have used the data from the service to understand and gauge the information-seeking pattern around public health topics, including cancer [66], antibiotics and probiotics [67], and COVID-19 [68]. For the current study, we used keywords such as “COVID vaccine”, “COVID cure”, “COVID vaccine side effects”, and “COVID 5G”. Given the intense interest in the abovementioned topics, we designed the questionnaire for Study 2. The analysis period was from 1 January 2021 to 30 June 2022.

This research aimed to describe the prevailing social, psychological, and digital factors to understand the vaccine hesitancy phenomena among adults. Study 2 employed a cross-sectional design vis-à-vis survey method for data collection from the target population of Internet users in Pakistan. Based on the study’s purpose, Internet users potentially exposed to COVID-19 vaccine-related content were the target population. This was necessary to investigate the possible influence of infodemics, conspiracy beliefs, and religious fatalism on vaccine hesitancy. Furthermore, this research also deciphers the role of demographic attributes in developing vaccine hesitancy. Through intensive literature mapping, leading factors were identified and investigated for greater demographic insight. To unfold these patterns of vaccine hesitancy among Pakistani nationals, a national representative sample of 2120 Internet users was collected through a triggered online survey method. The criteria for inclusion of the respondents were (1) ages 18 and above and (2) Internet users.

Two “filter” questions regarding the criteria delineated above were asked of the respondents. Those who responded and fulfilled the criteria were included in the survey. Given the above prognosis, an online questionnaire was administered using a Google form link. It was disseminated through posting on numerous social networking sites (i.e., Facebook and WhatsApp groups) to reach a large convenience sample. The response rate for this research is challenging to calculate as multiple digital platforms were used to disseminate and maximize the responses. An invitation to voluntary participation in this research was widely sent on digital platforms. Interested participants agreed and clicked through the Google form link read the guidelines, gave informed consent, and agreed to the ethical consideration involved in this research. An online form was used to ensure Internet users’ participation in the data collection. The invitation was repeated on popular social networking sites until the desired representative sample was collected. Using several online data collections using digital platforms were valuable to avoid selection biases. According to the literature [69], selection bias can remain an issue in a convenient and purposive sample due to the selection of the participants from easy to contact groups (e.g., peers or students) who can recruit others (e.g., general Internet users).

Furthermore, the selection bias emerged by recruiting a sample from a particular geographical location. However, this research used multiple digital platforms to reach Internet users and did not ask volunteers to collect data from a particular geographical location. This technique helps to reduce the selection bias as people from diverse backgrounds (please see demographics Table 1) participated in the survey.

According to the 2017 Census, the population of Pakistan was 207.68 million [70]. Pakistan had approximately 61.34 million Internet users in January 2021 [71]. To confirm the sample representation, we first implemented a Power analysis, which verified that a sample size greater than 2100 is a suitable sample size with effect size f = 0.461 and power 0.90 (*p* = 0.001) for a model with four variables. Second, Morgan’s sample size determination formula also validated that the sample size of 2100 can exhibit adequate generalization. Therefore, a minimum sample size of at least 2100 adult Internet users would have been necessary to examine the selected variables in the Internet users in Pakistan. Data from the 2120 adult Pakistani Internet users were collected from 15 March 2022 to 30 July 2022. The sample of 2120 is suitable and justifiable to represent the Internet users in Pakistan.

### 3.2. Instrumentation

The variables of infodemics, conspiracy beliefs, and religious fatalism items were extracted from the literature based on the significant Google Trends tool results. The prior items were adapted from the literature [7,72] and modified based on the results obtained by the Google Trends tools to ensure the alignment between the public search about the COVID-19 vaccine and their perception. Therefore, the researchers used the items to measure the infodemics using the predominant searches about the COVID-19 vaccines. In comparison, the variable of the conspiracy beliefs was measured through the five items modified version of the scale adapted from Bogart et al.’s work. The Helpless Inevitability Subscale of the Religious Health Fatalism Questionnaire (RHFQHI) by Nageeb et al. was modified to measure fatalistic beliefs [73]. Finally, the five items were modified from the Duong et al. work to measure Vaccine Hesitancy [74]. All variables were measured on a five-point Likert scale anchoring (1 = Strongly Disagree to 5 = Strongly Agree).

This research followed the guidelines for the content and face validation procedure. The content validation procedure is an established method to evaluate the extent to which an instrument has suitable and representative items to measure a construct [75]. In doing so, the questionnaire was sent out to the ten experts (e.g., academicians) with construct definitions, original and modified scales items and the purpose of the research. They were requested to give feedback on a scale of 1 to 4 with no neutral response. Their response was calculated using Lynn’s approach for the content validity rating formula. Followed by the content and face validity procedure, a pilot study was carried out using a sample of 50 university students. The pilot study results revealed Cronbach’s alphas of 0.89, 0.83, 0.86, and 0.91 for the infodemics, conspiracy beliefs, religious fatalism, and vaccine hesitancy, respectively, and satisfied proceeding with the data collection. The aggregate of the item modification was satisfactory and above the value of 0.66.

## 4. Results

### 4.1. Google Trends Tool Results for Study 1

**Figure 1:**
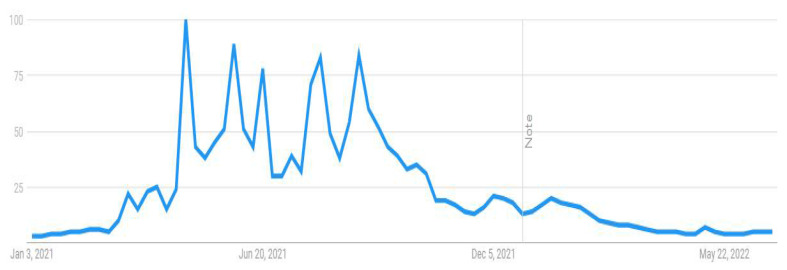
COVID-19 vaccine.

**Figure 2:**
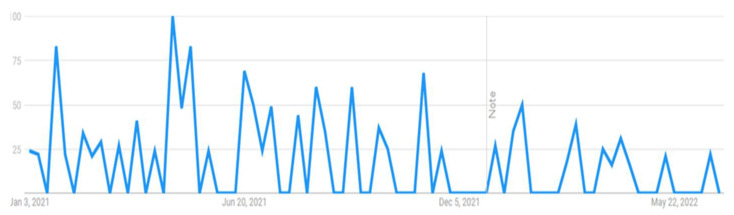
COVID-19 cure.

**Figure 3:**
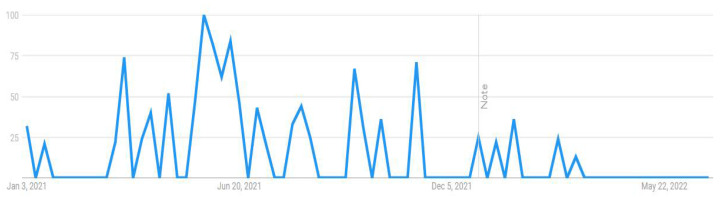
COVID-19 vaccine side effects.

**Figure 4:**
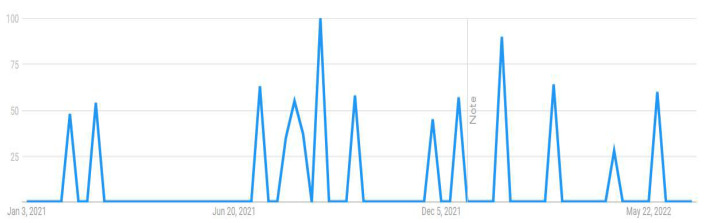
COVID-19 5G.

This research attempted to visualize the public searches about the COVID-19 vaccine from 1 January 2021 to 30 June 2022. Through the Google Trends feature, researchers can visualize the popularity of a particular search term on Google over time. This can help to gain insight into the patterns of public interest in the topic during a particular period with real-time frequencies. For example, when someone searches for a term on Trends, it can help him or her view a graph showing its popularity in (nearly) real time. Using the mouse’s cursor over the graph, one can see a number that reflects the term’s relative popularity based on the total number of searches conducted on Google. The numbers on the graph in Figure 1, Figure 2, Figure 3 and Figure 4 are not absolute search volume numbers but are presented on a scale from 0 to 100, with each point on the graph being divided by the highest point (100).

The numbers next to the search terms at the top of the graph represent the relative totals. Suppose the line on the graph is trending downward. In that case, it means that the search term’s relative popularity is decreasing, not necessarily that the total number of searches for the term is decreasing, but rather that its popularity, compared to other searches, is decreasing. The results of this study presented in the output from Google Trends data (see Figure 1, Figure 2, Figure 3 and Figure 4) indicated a strong interest among Internet users in Pakistan towards these topics, particularly during the peak of COVID-19. The results were used as a reference and contextual point for Study 2 and indicated that people have taken an interest in the COVID-19 vaccine-related issues and are vulnerable to the overwhelming information available online. The institutionalized information, such as journalistic practices through known and responsible media channels or health authorities, is mainly reliable. However, these results showed that people come across general Google searches, and there are greater possibilities that they are exposed to information from less reliable sources and can be misled by such misinformation. Therefore, a survey study was followed to analyze the public vaccine hesitancy, and its theoretically potential contributors were underpinned. The following section reports the results of the survey study.

### 4.2. Survey Results for Study 2: Demographic and Descriptive Statistics

A total of 2120 participants completed the survey. Of the total respondents, 1344 (63.4%) were males, and 776 (36.6%) were females, with a mean age of 34.5 (SD: ±6.23) years. The frequency of married participants was 1245 (58.7%), single participants 850 (41.1%), and divorced/widow participants 29 (1.4%). The respondents of illiterate were 307 (14.5%), primary school education respondents 289 (14.9%), secondary school education respondents 308 (14.5%), undergraduate level respondents 854 (40.3%), and postgraduate level respondents 362 (17.1%). The respondents of the lower class were 253 (11.9%), middle-class respondents 328 (15.5%), upper middle-class respondents 519 (24.5%), and upper-class respondents 1020 (48.1%). The detail of the demographics of participants can be seen in Table 1.

Before proceeding with the descriptive analysis, data normality was attained by deleting the outliers. The normality assumption was required for the analytical approach of structural equation modeling (hereafter SEM) used for this research. The SEM is a more robust approach for verifying the results’ theoretical factor structure, validities, and reliability. Furthermore, SEM permits researchers to conclude more authentic results and consider them the next generation of regression as it can assist in testing multiple factors prediction simultaneously. Therefore, this research employed univariate, bivariate, and multivariate outlier analysis to meet the data normality assumption. Next, the study employed descriptive statistics using SPSS 24.0 to verify possible associations between the variables of interest. During this phase, a total of 109 cases were deleted from the original data set of 2120, which is lower than the recommended tendency of deletion [76]. Therefore, a total of 2011 responses were used for further analysis procedures. The correlation analysis revealed that infodemics significantly correlated with vaccine hesitancy (r = 0.184, *p*-value = 0.001). Moreover, conspiracy belief (r = 0.283, *p*-value = 0.001) and religious fatalism (r = 0.179, *p*-value < 0.001) were significantly correlated with vaccine hesitancy, as shown in Table 2.

### 4.3. Confirmatory Factor Analysis

**Figure 5:**
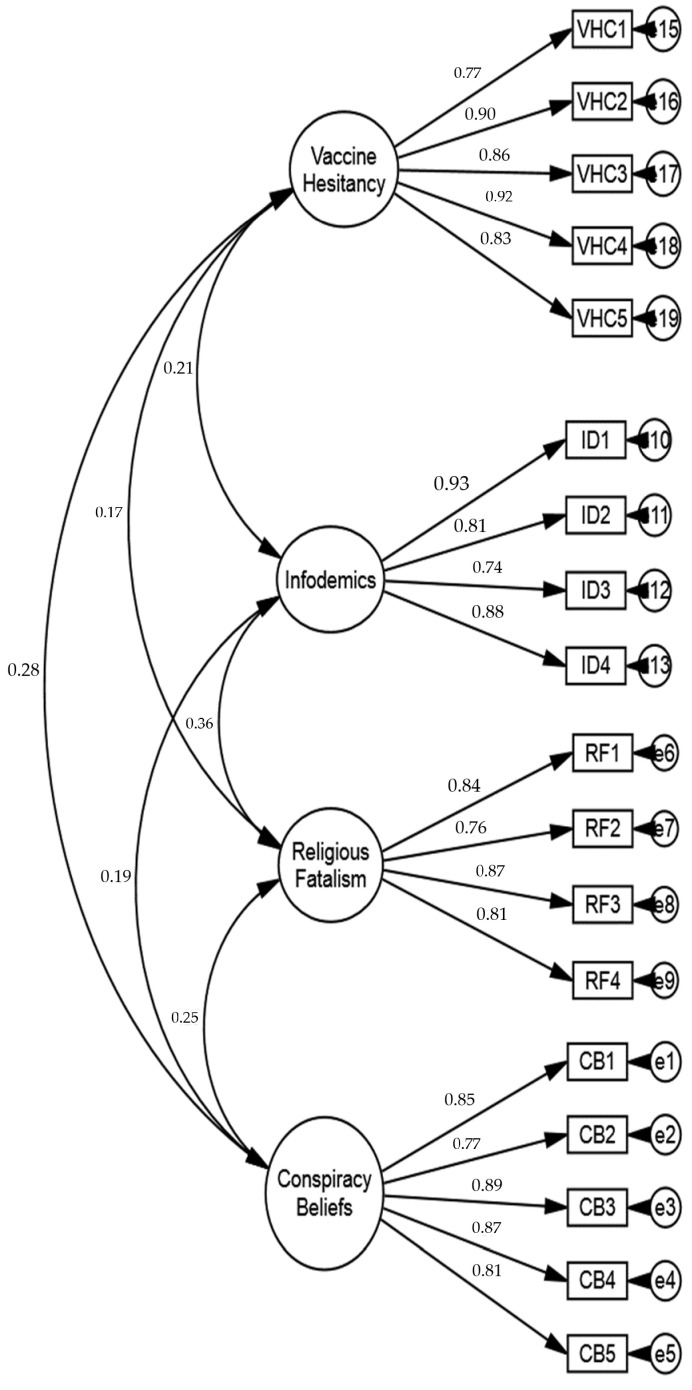
Measurement model.

The study employed the structural equation modeling technique to verify the (a) measurement model fitness, (b) convergent and discriminant validity, and (c) possibility of multidimensionality. To do so, all items were loaded on the parent constructs of infodemics, conspiracy beliefs, religious fatalism, and vaccine hesitancy to carry out the confirmatory factor analysis (hereafter CFA) using AMOS 24.0. The results of the CFA demonstrated that infodemics, conspiracy beliefs, religious fatalism, and vaccine hesitancy were empirically distinct (see Table 2). The six fit indices were evaluated to assess the goodness of the measurement model. The results revealed a good fit model after deletion of two items as; x^2^ = 1427, x^2^/df = 2.73 CFI = 0.96, GFI = 0.93, NFI = 0.93, TLI = 0.96, and RMSEA = 0.048 [77]. The item loadings are reported in Table 3 and Figure 5.

Next, the study proceeded with inferential statistics. Furthermore, the convergent and discriminant validities were evaluated using the Fornell–Larcker criterion method. The results of the CFA revealed satisfactory values for the Composite Reliability (CR) of each variable (see Table 4). Furthermore, Average Variance Extracted (AVE) was calculated, and the correlation between infodemics, conspiracy beliefs, religious fatalism, and vaccine hesitancy variables were lower than the square root values of each variable’s AVE.

### 4.4. Hypothesis Testing

**Figure 6:**
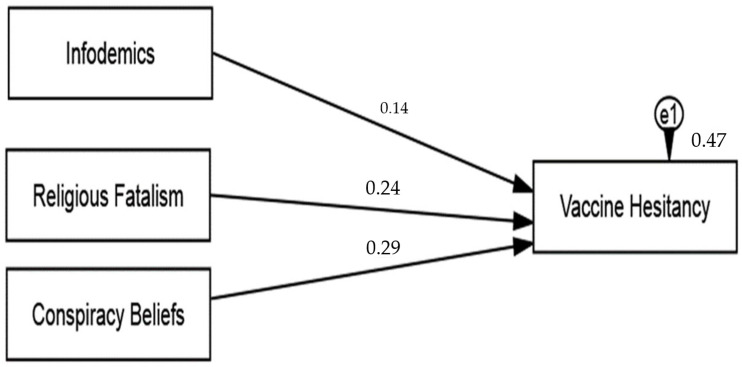
Path analysis.

This study postulated three hypotheses delineating the direct impact of infodemics (H1), conspiracy belief (H2), and religious fatalism (H3) on vaccine hesitancy. The path analysis was performed on AMOS 24.0 after computing the variables after the deletion of the items during CFA. The analysis results demonstrated that the infodemics significantly predict vaccine hesitancy (β = 0.14), in support of H1. The results also revealed that conspiracy beliefs significantly predict vaccine hesitancy (β = 0.29), and H2 was supported (see Table 5 and Figure 6).

The results also revealed that the conspiracy belief significantly predicts vaccine hesitancy (β = 0.24), and thus H2 was supported. Lastly, the path analysis verified the direct impact of religious fatalism on vaccine hesitancy, and H3 was also supported (see Table 5). A separate strategy is adopted and discussed in the next section for the moderation analysis.

### 4.5. Moderating Analysis

In this research, the moderating role of gender was postulated. This research followed Dawson’s approach [78] to verify the moderating influence of the categorical variables (e.g., gender). Dawson’s approach allows this research to empirically validate the interaction effect of gender in determining the association between infodemics and conspiracy belief with vaccine hesitancy. For evaluation, in this approach, two separate moderated multiple regression (MMR) analyses were employed for the infodemics and conspiracy belief interaction with the gender. To this end, the described gender by the participants was coded as a binary variable, male (X = 0) and female (X = 1). Therefore, in the analysis of this research, male serves as the reference group (e.g., the control group).

Secondly, the interaction term infodemics X gender (Male, X = 0, and female = 1, dummy implied) and control variables (e.g., income) were computed in the model. The results of the analysis suggested that gender moderates the association between infodemics and vaccine hesitancy significantly (see Table 6). The direct influence of the infodemics on vaccine hesitancy for the males was (gradient = 0.13), and (gradient = 0.19) for the females. These were significant and different and supported hypothesis 4(a). The results revealed that the infodemics resulted in a greater extent of vaccine hesitancy among the females. We concluded that females are more vulnerable to the inverse influences of the infodemics (see Table 6).

Lastly, the interaction term conspiracy beliefs X gender (Male, X = 0, and female = 1, dummy implied) and control variables (e.g., income) were computed in the model. The results of the analysis suggested that gender significantly moderates the association between conspiracy beliefs and vaccine hesitancy (see Table 6). Whereas the direct influence of the conspiracy beliefs on vaccine hesitancy for the males was (gradient = 0.21), the gradient for females was 0.34. The two were significant and different and supported hypothesis 4(a). The results revealed that conspiracy beliefs resulted in more vaccine hesitancy among females. We concluded that females are more vulnerable to the inverse influence of conspiracy beliefs.

## 5. Discussion

This study investigated popular search terms during the COVID-19 pandemic as reported on Google Trends. These terms revealed how popular certain search terms were during the pandemic which gives us an idea of what kind of information users wanted to learn at various points during the course of the pandemic. For example, charting the popularity of the search for vaccine side effects helps us understand vaccine hesitancy. Our research also examined infodemic overload, conspiracy beliefs, and religious fatalism regarding COVID-19 and the role of each in vaccine hesitancy. In addition, we investigated how gender moderates the relationship between infodemics and vaccine hesitancy and conspiracy belief with vaccine hesitancy. The study postulated four hypotheses. The evidence found in this study supported the four hypotheses, thus supporting the central proposition of this study that infodemics, conspiracy beliefs, and religious fatalism influence vaccine hesitancy. To restate, hypothesis H1 states that infodemics positively impact vaccine hesitancy towards COVID-19 (VHC) is supported. Likewise, H2 proposes that conspiracy beliefs positively impact VHC is also sustained. In a similar fashion, the assumption that religious fatalism positively impacts VHC is also validated. In addition, the findings of moderation analysis corroborate that gender moderates the relationship between infodemics and conspiracy beliefs and VHC. More succinctly, the findings from the moderation analysis supported the hypothesis that females are more susceptible to infodemics and conspiracy beliefs regarding their intentions about vaccination compared to males, who were found to be less vulnerable to the influence of infodemics and conspiracy beliefs abundant in the contemporary media ecology environment (Table 6).

### 5.1. Infodemic and Vaccine Hesitancy towards COVID-19

In the contemporary media ecological environment, infodemics are one of the significant issues that health authorities confront regarding vaccination campaigns. Our findings support H1 and validate that exposure to infodemics corresponds to vaccine hesitancy. Therefore, the greater the exposure to infodemics, the greater the vaccine hesitancy towards COVID-19 vaccines. These findings align with the previous literature [50]. As a result, the various forms of infodemics pose a challenge to healthcare campaigns and social and political systems around the globe. The COVID-19 pandemic saw a rise to a variety of infodemics such as misinformation, including rumors, myths, superstitions, fake news, and misleading content leading to mistrust in science [15] and ultimately affecting the acceptance of vaccines around the world. To increase the efficacy of communication campaigns to address challenges to healthcare systems around the globe, the regulation and some editorial control of user-generated content, such as blogs accessible using Google search, are indispensable to dealing with pandemics such as COVID-19 effectively.

### 5.2. Conspiracy Belief and Vaccine Hesitancy towards COVID-19

The public’s trust in vaccines is another worldwide challenge confronting health systems. Conspiracy beliefs cultivated due to conspiracy theories are a prominent factor in inducing vaccine hesitancy. Hypothesis H2 states that conspiracy beliefs positively impact VHC. These findings are consistent with the previous literature on conspiracy beliefs and VHC [52,53,54]. These beliefs are contributory factors among many others for posing a threat to the credibility of governments, science, and health institutions. As a result, anti-vaccination attitudes are being cultivated. To deal with conspiracy beliefs and minimize their influence, media literacy in general and digital media literacy in particular in the contemporary media world is one of the remedies. Digital media literacy enables us to deal with unregulated and opinionated content flooded on social media platforms responsible for inducing vaccine hesitancy. Moreover, a detailed examination of meanings and concepts associated with conspiracy beliefs may be helpful to assist scholars and health practitioners restore public trust in science and vaccines.

### 5.3. Religious Fatalism and Vaccine Hesitancy towards COVID-19

Our H3 supported that religious fatalism corresponds to vaccine hesitancy towards COVID-19. These findings support the results of previous studies validating that religious beliefs correspond to individuals’ health decisions [60]. The literature is replete with research reporting that several religious beliefs can adversely influence health behavior and reduce the public health response [61]. For instance, Muslims’ views of health are influenced by their feeling of religious fatalism, which includes the belief that “all is in Allah’s hands” and their perception of their powerlessness to prevent death when it is Allah’s will [79], and similar views on health contribute to a rise in Muslim populations vaccination hesitancy [80]. Similar to this, getting vaccinations were prevented in several prior non-COVID-19 instances in middle-income countries such as Malaysia in the cases of measles, mumps, and rubella (MMR). The religious rule against vaccines considered them to be “haram” (forbidden) due to the possible inclusion of ingredients originating from pigs [81]. A factor in Muslims’ growing hesitancy to get the COVID-19 vaccine may be the belief that vaccinations constitute a “medical assault” as well as concerns about the vaccines’ ingredients (such as whether they include pig gelatin) [82]. The COVID-19 vaccinations have been seen as a “Western scheme” to sterilize Muslim women in Asian nations such as Pakistan. Therefore, the public has generally been against vaccination [83].

### 5.4. Demographics

Demographic variables play a substantial role in the intention to take COVID-19-related precautionary measures. For instance, age significantly moderates vaccine hesitancy towards COVID-19 [84]. H4 of this study validated that females are more susceptible to infodemics and conspiracy beliefs than males. As a result, they show more vaccine hesitancy to COVID-19 than males. These results have verified that demographic attributes contribute to developing health responses. To this end, these results align with the previous studies [85] that suggested a critical role of demographic attributes in determining the health responses of people. Thus, demographic factors must be weighed when designing the strategic plan, particularly communicative actions that must be considered to educate vulnerable groups to respond to the health crisis efficiently. Previous research also found a similar trend in demographic disparity [86]. This research advances the understanding in this regard, and results have verified the crucial role of consideration of demographic attributes in managing a public health crisis. In short, strategic communication campaigns designed to minimize vaccine hesitancy must consider demographic variables such as gender to make them effective.

### 5.5. Managerial Implications

This study provides numerous managerial implications by delivering novel results regarding digital media usage and its influence on diverse demographic groups. This study used Google Trends to explore the search patterns of the public that have yet to be studied. We unfolded the critical aspect of what the public searches and usually pays attention to during a crisis. The policy makers can utilize the results of this research to modify the search engine optimization for valuable resources that the public can access in a future public health emergency. Moreover, the results of trends also give directions for better strategic management of communicative resources. For example, government and health managers can use Google Trends to find public digital media usage for other epidemics or endemics, such as polio vaccination. This exploration can give them guidelines to provide better content to educate the public about the adversities and actionable knowledge to counter public health-related challenges. The survey research has also provided lessons for health managers by unleashing demographic-related valuable information in a South Asian context. This information identified the most vulnerable communities and demographic groups policy makers can target to counter the health hazards and challenges.

From the strategic communication point of view, demographic information is a precious resource. Managers can plan targeted messages instead of general awareness campaigns to educate the most vulnerable demographic groups. For instance, the results of this research highlighted that women are more vulnerable to infodemics (overabundance of information) that lead to confusion. This data can help provide more information to women to decrease the chances of vaccine hesitancy not limited to COVID-19. Therefore, health managers can assist vulnerable communities by educating their communities and making them better prepared to combat health hazards in the future using several communicative means.

### 5.6. Limitations and Future Research

The current study employed the cross-sectional design to verify the adverse influence of infodemics, conspiracy beliefs, and religious fatalism on vaccine hesitancy. Coupled with the Google Trends study, it provides novel findings linking digital media usage patterns with possible adverse outcomes. However, this research has a few limitations; firstly, future studies can use a more sophisticated sampling approach owing to the research resources; this research used a feasible sampling technique for data collection. Secondly, this research has compared the influence of digital media usage. Instead, future research may tap social network usage with digital media usage and then merge the directions from these studies. Lastly, future studies can conduct experimental research to identify compelling online content. These studies can benefit the literature by providing insight into the strategic use of advantageous vs. adverse content to assist policy makers.

## 6. Conclusions

The COVID-19 pandemic saw an unprecedented increase of infodemics, conspiracy beliefs, misinformation, disinformation, conspiracy beliefs, and religious fatalism. These COVID-19-related packages of information distort facts about COVID-19. As a result, online discourse about COVID-19 polarizes [87] and creates more confusion over what is true and what is false. This study, therefore, suggested that literacy in general and media literacy vis-à-vis digital media literacy is an essential tool to combat infodemics, conspiracy beliefs, and religious fatalism. In addition, the study identified that females are more prone to infodemics and conspiracy beliefs widely circulated on digital media platforms. Therefore, strategic communication campaigns must pay attention to gender to effectively lessen the impact of the aforementioned facts. Hence, the study advances the understanding of the understudied facet of how digital media usage and potentially harmful factors such as infodemics are associated with each other to determine the public health response. Moreover, the results of the Google Trends can serve as resources to direct health managers to identify the domains of knowledge gaps in society. Moreover, it also provides insight into public information-seeking trends. Thus, governments and health planners can utilize this information to develop relevant materials and content for the public during or well before the expected health hazards. Furthermore, this research provides in-depth information about the critical factors of demographic attributes that can significantly benefit health managers’ strategic and communicative planning to combat a public health crisis. The results verified that identifying vulnerable demographic groups is a significant factor for better public preparedness. This is a significant contribution to the existing body of knowledge. Finally, the results describe the demographic disparity of the association between adversities such as infodemics and vaccine hesitancy. Therefore, the results emphasize the need for targeted communication campaigns instead of general messages and hint at the need to understand the importance of factors while planning communicative actions.

## Figures and Tables

**Table 1:** Demographic characteristics of the study participants.

Demographics		Mean	SD
Age (years)		34.5	12.0
		Frequency	Percentage
Gender	Male	1344	63.4%
	Female	776	36.6%
Marita status	Single	850	41.1%
	Married	1245	58.7%
	Divorced	29	1.4%
Education	Primary	307	14.5%
	Secondary	289	14.9%
	High school	308	14.5%
	Undergraduate	854	40.3%
	Postgraduate	362	17.1%
Family Income	Lower class	253	11.9%
	Middle class	328	15.5%
	Upper MC	519	24.5%
	Upper class	1020	48.1%

**Table 2:** Correlation Statistics.

Variables	Infodemics Status	Conspiracy Belief	Religious Fatalism	Vaccine Hesitancy
Infodemics	1			
Conspiracy belief	0.130	1		
Religious fatalism	0.344	0.255	1	
Vaccine hesitancy	0.184	0.283	0.179	1

**Table 3:** Item standardized weights.

Variables	Estimate
Infodemics	
ID1	0.93
ID2	0.81
ID3	0.74
ID4	0.88
ID5	0.47 *
Conspiracy Beliefs	
CB1	0.85
CB2	0.77
CB3	0.89
CB4	0.87
CB5	0.81
CB6	0.39 *
Religious Fatalism	
RF1	0.84
RF2	0.76
RF3	0.87
RF4	0.81
Vaccine Hesitancy	
VHC1	0.77
VHC2	0.90
VHC3	0.86
VHC4	0.92
VHC5	0.83

* Item removed.

**Table 4:** Validity statistics.

Variables	CR	AVE	VHC	CB	ID	RF
Vaccine Hesitancy	0.932	0.736	0.877			
Conspiracy Beliefs	0.922	0.704	0.281	0.855		
Infodemics	0.907	0.710	0.206	0.187	0.863	
Religious Fatalism	0.891	0.674	0.175	0.254	0.356	0.958

**Table 5:** Standardized regression weights.

			*β*	T-Value	*p*	Hypotheses
Infodemics- > Vaccine Hesitancy			0.14	3.81	0.001	H1 Accepted
Conspiracy beliefs- > Vaccine Hesitancy			0.29	7.91	0.001	H2 Accepted
Religious fatalism- > Vaccine Hesitancy			0.24	4.45	0.001	H3 Accepted

**Table 6:** Moderating statistics with gradients findings.

Moderation Effects	*β*	Standard Bootstrap Outcomes					Hypothesis
		T Value	*p* Value	Male Gradient	Female Gradient	Durbin Watsons Test	
(Gender X Infodemics) → VH	0.18	3.12	0.01	0.13	0.19	1.89	H4(a) Accepted
(Gender X CB) → VH	0.26	4.87	0.03	0.21	0.34	2.05	H4(b) Accepted

## Data Availability

The data supporting this study’s findings are available from the corresponding author upon reasonable request due to ethical and privacy restrictions.
